# “I am resting but rest less well with you.” The moderating effect of anxious attachment style on alpha power during EEG resting state in a social context

**DOI:** 10.3389/fnhum.2014.00486

**Published:** 2014-07-11

**Authors:** Willem J. M. I. Verbeke, Rumen Pozharliev, Jan W. Van Strien, Frank Belschak, Richard P. Bagozzi

**Affiliations:** ^1^Department of Marketing, Erasmus School of Economics, Erasmus University RotterdamRotterdam, Netherlands; ^2^Department Brain and Cognition, Erasmus Institute of Psychology, Erasmus University RotterdamRotterdam, Netherlands; ^3^Department of Faculty of Economics and Business; Section HRM and Organisational Behaviour, Amsterdam Business School, University of AmsterdamAmsterdam, Netherlands; ^4^Department of Marketing, Ross School of Business, University of MichiganAnn Arbor, MI, USA

**Keywords:** EEG, adult attachment style, alpha frequency band, resting state, social context

## Abstract

We took EEG recordings to measure task-free resting-state cortical brain activity in 35 participants under two conditions, alone (A) or together (T). We also investigated whether psychological attachment styles shape human cortical activity differently in these two settings. The results indicate that social context matters and that participants' cortical activity is moderated by the anxious, but not avoidant attachment style. We found enhanced alpha, beta and theta band activity in the T rather than the A resting-state condition, which was more pronounced in posterior brain regions. We further found a positive correlation between anxious attachment style and enhanced alpha power in the T vs. A condition over frontal and parietal scalp regions. There was no significant correlation between the absolute powers registered in the other two frequency bands and the participants' anxious attachment style.

## Introduction

In neuroscience there is ongoing debate as to what exactly resting-state (awake) activity entails. Some studies suggest that resting state refers to introspective processes such as mind wandering (McKiernan et al., 2006; Mason et al., 2007). Other studies propose that resting state refers to self-reflection (Greicius and Menon, 2004; Buckner and Carroll, 2007; Moran et al., 2013), which could involve thinking of possible social interactions with others (Mitchell, 2006; Rilling et al., 2008).

Nevertheless, EEG oscillations, which might take place in different frequency ranges, are extremely structured and systematic even in the absence of specific goal-directed (resting-state) tasks. In EEG, alpha band activity is a well-known type of brain oscillation consistently observed during resting state and usually more pronounced over the parietal-occipital cortex (Scheeringa et al., 2012). However, despite more than 80 years of human EEG research, the exact functional role of alpha oscillations remains an open question. The amount of alpha activity in a given brain state (e.g., resting state vs. attention during a task) is commonly regarded as an inverse index of cortical excitability (Adrian and Matthews, 1934; Mo et al., 2013). According to one of the most generally accepted theories, enhanced alpha power reflects an active mechanism for inhibitory top-down control (Klimesch et al., 2007). Sauseng et al. (2005) and Cooper et al. (2006) provide evidence for this theory, reporting enhanced alpha synchronization in tasks requiring internally oriented attention, such as mental imagery. This perspective suggests that enhanced alpha power in the posterior brain region is associated with decreased connectivity with other tightly connected brain regions (Scheeringa et al., 2012). One possible functional interpretation of this empirical evidence is that increased alpha power over posterior regions during tasks requiring internal attention might inhibit visual activity in order to preserve internal processes, such as self-referencing, from being disturbed by possible external sensory information (Mo et al., 2013). Most importantly, however, past research consistently reports strong relationships between alpha power and tonic alertness. Enhanced alpha band activity has been related to tonic maintenance of attentional resources (Dockree et al., 2007). Moreover, decreases in ongoing alpha power are associated with impaired behavioral performance (Makeig and Jung, 1995).

Research shows evidence for an existing constellation of brain areas known as the default mode network (DMN), including the medial prefrontal cortex and medial parietal cortex, which are more active during task-free resting state than during goal-directed tasks (Raichle et al., 2001; McKiernan et al., 2003; Fox et al., 2005; Moran et al., 2013). The DMN is believed to reflect processes such as task-independent introspection and self-reflection, which usually occur during resting state (Buckner et al., 2008; Christoff et al., 2009). Interestingly, DMN activity corresponds to EEG power variations in different frequency bands. For instance, frontal and parietal DMN activity is associated with high alpha and beta band activity (Mantini et al., 2007; Hlinka et al., 2010). Jann et al. (2009) reported enhanced alpha and beta oscillation related to DMN activity. All this research suggests the existence of a close relationship between the behavior of DMN and alpha band activity, especially in the resting state.

The level of blood-oxygen-level dependent (BOLD) activity in the DMN is strongly related to alpha power modulations over posterior brain regions. However, simultaneous EEG-fMRI sessions are required to establish this relationship. For the sake of simplicity and because of the exploratory character of our study, we seek to examine to what extent activation in the different EEG frequency bands is modulated by changes in the social context in which the task-free resting state condition is carried out. Thus, we focus exclusively on electrophysiological changes of brain activity and leave for future research more complex hemodynamic responses. Based on the empirical and theoretical evidence reported so far, we expect alpha band activity during resting state to be a reliable EEG indicator of tonic alertness or processes requiring internally oriented attention, such as mental imagery and self-reflection (Sadaghiani et al., 2010).

To date, resting state has been generally examined in a traditional single person fashion. Yet, researchers have recently recognized the need for a more socially valid approach to unraveling certain distinct patterns of ongoing brain activity. This reasoning resonates with the recent work of Schilbach et al. (2008) showing that resting state reflects people's self-conscious processes. Nevertheless, being self-conscious is socially situated and context dependent, which means that people often have to reason how they differ and relate to others depending on the social context. Schilbach et al. (2008) did not study resting state in a social context or variations in social conditions. It is perhaps questionable to expect that changes in social context, which might range from performing a goal-directed task to simply being awake in a resting state, sitting passively with another person, might modulate human brain activity in exactly the same way as occurs when one is alone. Many biological and physiological regulators may influence brain responses in social contexts. For example, in interpersonal relationships, human adults develop somewhat stable, trait-like individual differences commonly referred to as attachment styles (Mikulincer and Shaver, 2010). Considering the task-free resting-state condition, the main topic of our research, we believe that attachment systems might mediate ongoing brain activity in relation to changes in the social environment. Therefore, in our study, we decide to examine the resting state using EEG methods, with particular focus on whether human attachment styles affect resting state brain activations in different social contexts. Our paper builds upon Vrtička and Vuilleumier (2012, p. 14) proposal that the study of neural correlates of attachment styles should investigate people in a social context rather than in social isolation.

Attachment systems play a relevant role in the way people relate to other people, especially in cases of need. The attachment system is based on basic needs such as protection and security, and is usually activated in situations involving threat or distress. From a neurological perspective, attachment is an evolutionary hardwired system that is shaped during contact with early caretakers. It consists of reflexive avoidant and approach systems (affective evaluation network) that operate in a push-pull manner: e.g., under stress, people reflexively avoid negative stimuli and seek proximity with others to experience the neuroception of safety (Vrtička and Vuilleumier, 2012). Research shows that the early caretaker's reactions to their child's proximity seeking behavior develops into a person's working model of relationships with other people and produces “mental simulations of how other people would respond to their proximity seeking behavior” (Mikulincer and Shaver, 2010). This type of social mentalization tunes the reflexive attachment system to requirements of the social environment (Vrtička and Vuilleumier, 2012).

Past behavioral research with mother-child relationships and adult relationships reveals that trait-like individual differences exist in two separate styles: namely, attachment insecurity and attachment security. Attachment insecurity occurs along two independent axes: attachment anxiety and attachment avoidance. Overall, three types of attachment styles have evolved: secure, avoidant, and anxious. Under the first, securely attached individuals handle stress by seeking support from trusted people or by calling upon mental representations of support received in the past (Mikulincer and Shaver, 2003). The second, avoidant attached style, is marked by a certain amount of self-reliance in social behavior. Avoidant individuals tend to deactivate their attachment system in socially stressful situations, and do not experience much negative feelings when rejected. Thus, their proximity seeking behavior is relatively low. Finally, anxious individuals are highly sensitive to both rejection and acceptance in socially stressful situations, which is manifested in hyperactivation of their attachment style when engaged interpersonally with others. However, despite extensive clinical and social psychological research, very little is known about the way in which attachment styles are represented in the brain. The few studies that have attempted to establish relationships between neural systems and attachment styles have mainly used indirect measures of brain activity such as fMRI. Generally, neuroimaging studies examine attachment styles in relation to processing emotional information or memory processes (Vrtička et al., 2008; Donges et al., 2012). Because of its high temporal resolution, ERP research has much promise for the study of attachment styles. Nevertheless, in most cases to date, ERP research has been limited to emotional and memory processes of individuals in non-social contexts (Escobar et al., 2013). In our study, we examine the social context in which the relationship between attachment style and neural activity occurs. We believe that the study of social behavior in the absence of any goal-directed task or redundant external information provides a clear and well-defined experimental setting in which to study the possible influence of attachment style on brain activity.

Resting state *per se* is not well-understood, especially in connection with changes in social context. However, some researchers argue that during resting state people reflect on whom they might interact with (e.g., Schilbach et al., 2008). This process involves a mental simulation of how they would respond to other people in a specific situation or how this other person would respond to them. A closer look at this social simulation implies that, for one individual to reflect upon how he would respond to another individual in a specific social situation, he needs to mentally elaborate on who that other person is (Schilbach et al., 2008).

One approach in this regard is the following. Resting with another person nearby, compared to resting completely alone, can be conceived as a social situation low on interpersonal feedback. Functioning in this minimal social context might activate the reflexive avoidant and approach system (affective evaluation network): e.g., “I am feeling uncertain about this minimal feedback.” Equally, such a situation is likely to provoke mental state representations (self-reflection) in relation to the other person: e.g., thoughts might occur such as “Why does this person not give me any feedback?” We know of only one study that has investigated the relationship between attachment styles and a person's non-involvement in a task. Sloan et al. (2007, p. 4) showed that during sleep, anxious attached people exhibit an alpha power anomaly, indicating that attachment anxiety is a marker of hypervigilance that increases individuals' sensitivity to harmful stimuli even during sleep.

As attachment styles reflect differences in mental simulation regarding their approach to interaction with others, for anxious attached people in a resting state, sitting passively beside each other, we can ask, if higher levels of tonic alertness will occur and be reflected in hyperactivation of their attachment style. In other words, could low social feedback in a resting state with another person be anxiety provoking, especially for people with an anxious attachment style, and might it in turn evoke mental simulations about the other person's judgments (e.g., “What does this other person think of me?”).

On the other hand, we do not expect a similar process to occur for avoidant attached people because they are relatively insensitive to feedback from other people. Avoidant attached people generally prefer situations where they do not have to socialize or relate to others extensively. Hence, we do not expect that the alpha power will be moderated significantly by individuals' scores on avoidant attachment.

We investigated the relationship between cortical brain oscillations occurring in different frequency bands and subjects' anxious and avoidance attachment styles, measured with psychological scales, by recording and comparing EEG data from two types of task-free resting state sessions: namely, a conventional A session where the participant is alone and a less conventional T session where the participant is together with another subject.

## Materials and methods

### Subjects

Forty healthy female undergraduates from a Dutch University, ranging in age from 18 to 26 years (Age *M* = 22.07, *SD* = 2.09), took part in this study. Participants enrolled in the experiment in exchange for educational credit. All participants had normal or corrected-to-normal vision. Informed consent was obtained at the beginning of the experiment. Five participants were excluded from the analysis because of an excessively high percentage of artifacts (using a criterion of 75% or less artifact-free epochs). Thus, we analyzed electrophysiological responses (EEG) and attachment style data from 35 participants.

### Questionnaire

After the EEG recording sessions, participants completed a questionnaire on attachment styles developed by Brennan et al. (1998). Three anxious attachment items were used in a 7-point Likert scale with “very untrue of me/very true of me” as end-points and “neutral” as a mid-point. For the present sample, total scores of the three items on the anxious attachment scale ranged from 5.0 to 21.0 (*M* = 10.92, *SD* = 3.54), with Cronbach's α equal to 0.61 (see Appendix 1). Two avoidant attachment items (reversed coded) were used with the same 7-point Likert scale as used for anxious attachment. Total scores on the avoidant attachment scale ranged from 6.0 to 14.0 (*M* = 10.45, *SD* = 2.22), with Cronbach's α equal to 0.81 (see Appendix 2).

### Procedure

The experiment was conducted in two sessions (A and T conditions). For the A condition, EEG recordings were collected from participants sitting isolated in a dimly lit EEG laboratory. For the T condition, two participants sat together in the same EEG lab. Participants sat in comfortable chairs approximately 100 cm away from, and at eye level with a 40 × 30 cm IIyama PC computer screen. In the T condition, the participants sat beside each other, both facing the computer screen. The order of the A and T conditions was counterbalanced. Participants interacted with each other during the installation of the EEG caps and in the period between the A condition and T condition sessions.

In both conditions, participants were shown a white fixation cross for 2 min, which was presented centrally on the computer screen using E-prime presentation software (Psychology Software Tools, Inc.). To reduce the number of EEG artifacts caused by eye movement, participants were instructed to relax and reduce blinking and other ocular movements during the experimental sessions.

### Electrophysiological recordings and analysis

The electroencephalogram (EEG) was recorded continuously from 32 active Ag/AgCI electrode sites using a BioSemi 32-channel elastic head cap with standard international 10–20 system layout. In the T condition, EEG was recorded with two identical 32-channel EEG caps. Each cap signal was acquired from two separate, identical amplifiers (BioSemi Active-Two system AD-box) that were connected to each other and the same computer with optical cable. Flat-type active electrodes were attached to the right and left mastoids. Electrodes located on the outer canthi of each eye, as well as below and above the left eye, were attached to measure bipolar horizontal and vertical EOG activity. In addition, an active pin-type electrode (CMS, common mode sense) and a passive pin-type electrode (DRL, driven right leg) were used to compose a feedback loop for amplifier reference. Online, EEG was digitized at a sampling rate of 512 Hz, 24-bit A/D conversion. Offline, we changed the sampling rate to 256 Hz.

Further offline processing was performed with Brain Vision Analyzer (Brain Products GmbH, Germany; www.brainproducts.com). Offline, the EEG signals were re-referenced to the average of the left and right mastoids. EEG data were band-pass filtered between 0.1 and 100 Hz. Artifacts caused by ocular movements were removed by applying Independent Component Analysis (ICA) with Brain Vision Analyzer (for more details see Brain Products GmbH, Germany; www.brainproducts.com). Band rejection filtering for 50 Hz (notch filter) was used to eliminate interference from the electricity network. After the ICA correction procedure, EEG signals were subjected to segmentation (2000 ms) and artifact-rejection processing. The artifact-rejection method consisted of excluding epochs with large amplitude (over ± 100 μV). Additionally, two experienced EEG researchers (blind to the stimulation condition) screened the EEG recordings for residual contamination of the EEG epochs due to eye or muscle artifacts. As a result, only epochs (2000 ms) completely free from artifacts were considered for the following spectral analyses.

In both studied conditions (A and T), the 2-min resting state EEG data were segmented and analyzed in 2000 ms epochs. This process resulted in 60 epochs per condition, of which some 55 valid epochs in the A and 54 valid epochs in the T condition across the 35 subjects, on average, were subjected to further spectral analysis. Each set of artifact-free EEG data (2000 ms epochs) was subjected to fast Fourier Transform (FFT) analysis with a 10% Hanning window, performed by Brain Vision Analyzer (Brain Products GmbH, Germany; www.brainproducts.com). To ensure an adequate signal-to-noise ratio of the EEG data, at least 45 artifact-free segments were required from each subject (for each condition) for fast Fourier transformation and power spectral analysis. Absolute EEG band power (μV^2^) for each of the selected scalp areas, Frontal (F3, Fz, F4), Central (C3, Cz, C4), Parietal (P3, Pz, P4), and Occipital (O1, Oz, O2) was calculated for Theta (4–8 Hz), Alpha (8–12 Hz), Beta (12–25 Hz), and Gamma (30–40 Hz) frequency bands which were defined based on a conventional EEG sense (e.g., Jacobs and Lubar, 1989; Hotz et al., 2000; Diego et al., 2004). After the FFT procedure, the artifact-free epochs were averaged for each A and T condition separately.

### Statistical analysis

The following electrodes were used for the data analysis: Frontal (F3, Fz, F4), Central (C3, Cz, C4), Parietal (P3, Pz, P4), and Occipital (O1, Oz, O2). For the group comparisons we employed a mixed-design analysis of variance (ANOVA), with Condition (Alone, Together), Caudality (Frontal, Central, Parietal, Occipital), Laterality (Left, Middle, Right), and Frequency (Theta, Alpha, Beta, Gamma) as within-subject factors and Anxious Attachment score (Low, High) as a between-subject factor for each frequency band. In this approach, the anxious attachment score was used as a grouping variable by means of a median split. The median-split approach allows a clear presentation of the repeated-measures results in both groups but has the statistical disadvantage that it may reduce power and lose information (MacCallum et al., 2002). Therefore, we also performed correlational analyses using the anxious attachment score as a continuous variable. For the ANOVAs, we checked multivariate normal distribution with the Mauchly sphericity test, and applied the Greenhouse-Geisser correction, when appropriate. A *p*-value of <0.05 was considered significant (Keeser et al., 2011). Significant interaction effects were followed by paired-sample *t*-tests. Bonferroni correction was implemented to adjust for multiple comparisons. Statistics were analyzed with the IBM SPSS 13.0 software (Statistical Package for Social Sciences, SPSS Inc., Chicago, IL).

## Results

### Behavioral results

Based on the attachment style scores, derived from the questionnaires, the 35 participants were assigned to high (HA) or low (LA) anxious attachment. More precisely, based on median-split approach 17 participants were assigned to HA group (above vs. below median scores = 11.00) and 18 to the LA group. The median split resulted in the following means for the HA group (*M* = 13.94; *SD* = 2.41) and LA group (*M* = 8.16; *SD* = 1.65). The order in which participants from different anxious attachment groups started the A vs. T condition was counterbalanced. Nine HA group participants started the EEG experiment with A condition, while 10 from the LA group started with T condition.

For the avoidance attachment style 17 participants were again assigned to high (HAV) and 18 participants to low (LAV) avoidant attachment groups (based on above vs. below median scores = 11.00). The median split resulted in the following means for the HAV group (*M* = 8.30; *SD* = 1.31) and LAV group (*M* = 12.23; *SD* = 0.96). Again, as for the anxious attachment style, the order in which participants from different avoidance attachment groups started the A vs. T condition was counterbalanced.

Pairwise A vs. T contrasts indicated that there was no significant difference between the number of eye blinks in A condition (*M* = 38.37; *SD* = 24.83) compared to T condition (*M* = 41.00; *SD* = 22.95), [*t*_(34)_ = −0.838, *p* = 0.408]. Pearson correlation revealed that the anxious attachment score did not correlate significantly with the number of eye blinks in A (*r* = 0.12, *p* = 0.483) or T (*r* = −0.07, *p* = 0.656) conditions. Avoidance attachment score also did not correlate significantly with the number of eye blinks in A (*r* = 0.05, *p* = 0.736) or T (*r* = 0.04, *p* = 0.778) conditions. Next, we tested whether the anxious attachment groups (HA and LA) have different avoidance attachment scores. A *t*-test indicated that there was no significant difference on avoidance attachment between HA (*M* = 10.88; *SD* = 2.26) and LA (*M* = 9.77; *SD* = 2.23) groups, *p* = 0.572. Finally, there was no significant correlation between anxious and avoidance attachment scales (*r* = 0.13, *p* = 0.427).

### Electrophysiological results

First, we tested whether there was a difference between HA and LA attachment groups with respect to the number of artifact-free epochs used for the electrophysiological analysis. *T*-test on the number of artifact-free epochs from the A condition revealed that there was no significant difference between the HA (*M* = 55.47; *SD* = 4.12) and LA (*M* = 54.83; *SD* = 4.61) attachment groups, *p* = 0.646. The same absence of significant difference was also found between HA (*M* = 54.23; *SD* = 5.03) and LA (*M* = 53.33; *SD* = 5.58) attachment groups, *p* = 0.314 with respect to the number of artifact-free epochs in T condition. Finally, pairwise A vs. T contrasts indicated no significant difference between the number of artifact-free epochs in A condition (*M* = 55.14; *SD* = 4.33) compared to T condition (*M* = 53.77; *SD* = 5.26), *p* = 0.161.

Repeated-measures ANOVA with Condition (Alone, Together), Caudality (Frontal, Central, Parietal, Occipital), Laterality (Left, Middle, Right), and Frequency (Theta, Alpha, Beta, Gamma) as within-subject factors and Anxious Attachment (Low, High) as between-subject factor on absolute EEG power revealed significant main effects for Condition [*F*_(1, 33)_ = 7.66, *p* = 0.009], Caudality [*F*_(3, 99)_ = 4.87, *p* = 0.007] and Frequency [*F*_(3, 99)_ = 84.57, *p* < 0.001]. However, these main effects were qualified by second-order interactions of Condition × Anxious attachment [*F*_(1.33)_ = 15.75, *p* < 0.001], Condition × Frequency [*F*_(3.99)_ = 5.41, *p* = 0.008], Frequency × Caudality [*F*_(9.297)_ = 20.56, *p* < 0.001] and by a third order interaction of Condition × Frequency × Anxious attachment [*F*_(3, 99)_ = 5.68, *p* = 0.006]. This third order interaction was further investigated by separate Condition × Anxious attachment ANOVAs for each frequency band.

**Figure 1:**
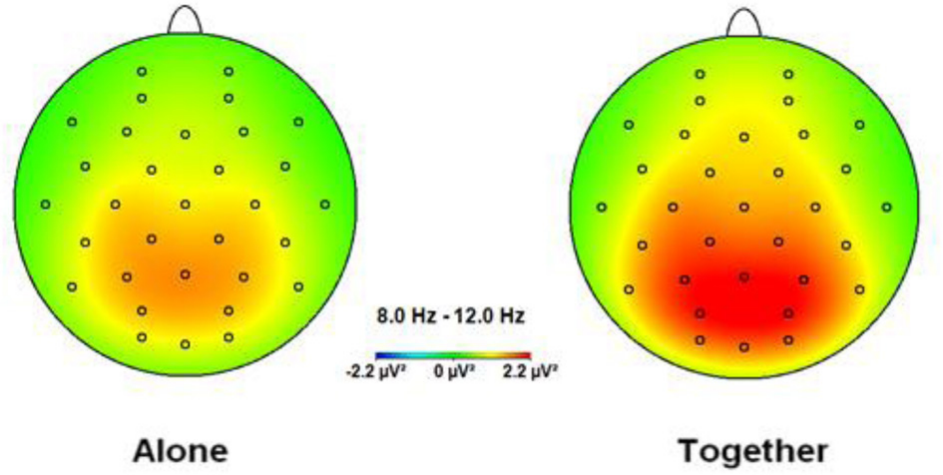
**Topographic spectral mapping of absolute EEG alpha power in alone vs. together conditions**. The increase in alpha power, marked strongly over the occipital-parietal cortex, is shown from the A to T condition.

**Figure 2:**
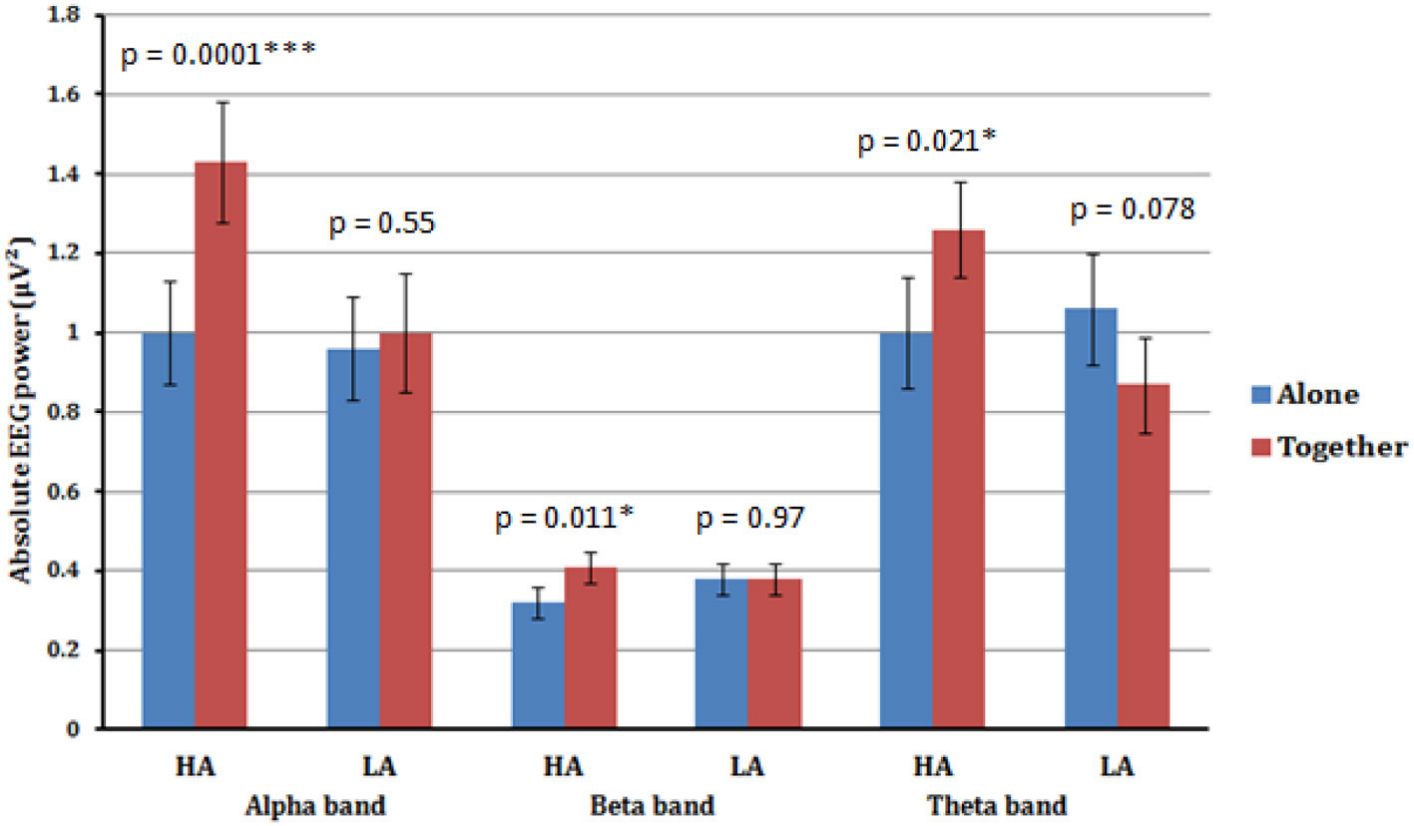
**Estimated marginal means (an average value from all four scalp areas) for alpha, beta, and theta frequency bands of high anxious (HA) and low anxious (LA) attached participants in A (blue) vs. T (red) conditions**. Significant increase in alpha, beta, and theta absolute powers from A to T condition is shown for high anxious (HA) attached participants. No significant difference across all frequency bands was detected between A and T conditions for low anxious (LA) attached participants.

The ANOVA for the alpha band showed a significant main effect for Condition [*F*_(1.33)_ = 15.13, *p* < 0.001], qualified by a significant interaction between Condition × Anxious attachment [*F*_(1.33)_ = 9.93, *p* = 0.003]. The EEG alpha power was significantly lower in the A condition (*M* = 0.98; *SD* = 0.53) compared to the T condition (*M* = 1.22; *SD* = 0.64) (Figure 1). More precisely, we detected a significant difference between the A condition (*M* = 1.00; *SD* = 0.52) and the T condition (*M* = 1.43; *SD* = 0.70) for the HA group, [*t*_(16)_ = 4.41, *p* = 0.0001] (Figure 2). No significant difference was detected when we compared the A condition (*M* = 0.96; *SD* = 0.56) and the T condition (*M* = 1.00; *SD* = 0.52) for the LA group, *p* = 0.55 (Figure 2). In addition, we report significant main effect for Caudality [*F*_(3.99)_ = 12.69, *p* < 0.001], with highest alpha value over parietal scalp areas (*M* = 1.34; *SD* = 0.76) and lowest alpha power over frontal areas (*M* = 0.83; *SD* = 0.41). However, this Caudality effect was not qualified by any significant interaction.

A significant main effect for Condition [*F*_(1.33)_ = 4.48, *p* < 0.042], qualified by a significant interaction between Condition × Anxious attachment [*F*_(1.33)_ = 4.31, *p* = 0.046] was also found in the beta frequency band. Again we detected a significant difference between the A condition (*M* = 0.32; *SD* = 0.11) and the T condition (*M* = 0.41; *SD* = 0.14) for the HA group, [*t*_(16)_ = 2.86, *p* = 0.011]. Additionally, there was no significant difference between the A condition (*M* = 0.38; *SD* = 0.20) and the T condition (*M* = 0.38; *SD* = 0.16) for the LA group, with *p* = 0.976 (Figure 2).

A significant interaction between Condition × Anxious attachment [*F*_(1.33)_ = 9.72, *p* = 0.004] was also found in theta frequency band. Again we detected a significant difference between the A condition (*M* = 1.00; *SD* = 0.44) and the T condition (*M* = 1.26; *SD* = 0.54) for the HA group, [*t*_(16)_ = 2.56, *p* = 0.021]. Additionally, there was no significant difference between the A condition (*M* = 1.06; *SD* = 0.66) and the T condition (*M* = 0.87; *SD* = 0.44) for the LA group, with *p* = 0.078 (Figure 2). No significant interaction between Condition and Anxious attachment was detected in the gamma frequency band.

Repeated-measures ANOVA with Condition (Alone, Together), Caudality (Frontal, Central, Parietal, Occipital), Laterality (Left, Middle, Right), and Frequency (Theta, Alpha, Beta, Gamma) as within-subject factors and Avoidance attachment (Low, High) as a between-subjects factor on the absolute EEG power did not reveal any significant interaction effects of Avoidance attachments with Condition, Electrode, or Frequency.

### Correlational analysis

**Figure 3:**
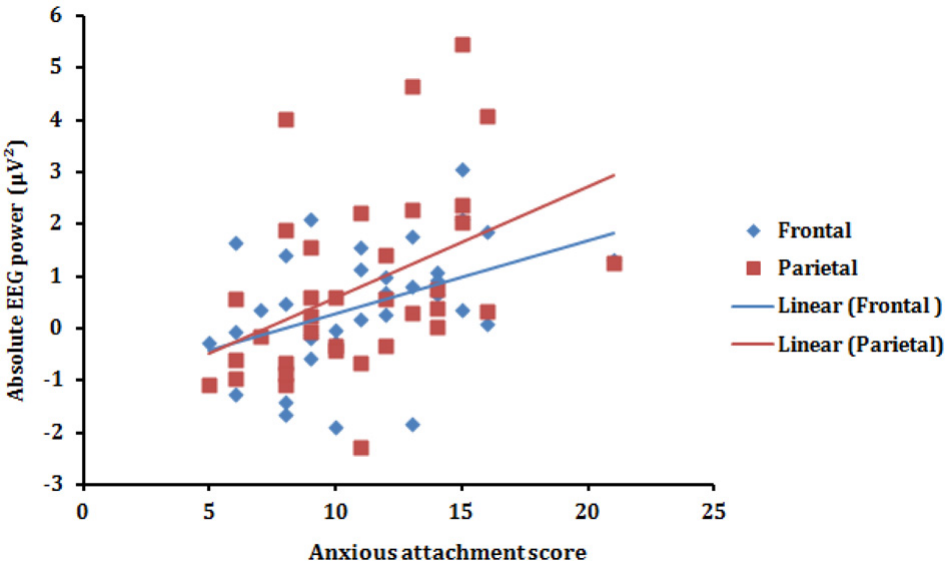
**Scatterplot (with regression lines) of the anxious attachment score and the absolute alpha power (T minus A power) presented in the frontal (blue) and parietal (red) scalp areas**.

To further explore the association between alpha synchronization in the T vs. A condition, we computed the correlation between the Anxious attachment score and the Condition effect for each of the four (Frontal, Central, Parietal, and Occipital) scalp areas (T minus A power). Pearson correlation revealed that anxious attachment score correlated significantly with alpha synchronization in the Frontal (*r* = 0.42, *p* = 0.011) and Parietal (*r* = 0.44, *p* = 0.009) scalp areas (Figure 3). This positive correlation between anxious attachment score and alpha power, especially in the posterior scalp locations, conforms with the results reported in the alpha band from ANOVA analysis. However, there were no significant correlations between anxious attachment scores and condition effect in the other EEG frequency bands.

## Discussion

As Vrtička and Vuilleumier (2012) recommend, the neural correlates of human attachment styles should be studied in a social context rather than in isolation, where the latter has been the typical practice in EEG studies on attachment to date (e.g., Sloan et al., 2007; Zilber et al., 2007; Zhang et al., 2008). Building upon Vrtička and Vuilleumier (2012) insights, our main goal was to investigate (a) the spatial distribution of EEG spectral powers when people are in A vs. T resting-state condition, (b) how these spectral powers vary between the A and T conditions, and (c) whether variations are shaped by the participants' anxious and avoidant attachment styles.

The result of our study clearly shows that participants experience enhanced alpha, beta and theta power when they are in the resting-state session together with another person compared to when they are alone. Most importantly, however, this result occurred only for high anxious attachment participants. No significant differences between the two resting-state sessions were found for the low anxious participants across all frequency bands studied. However, correlational analysis shows that this enhanced alpha power from A to T condition was associated with the participant's anxious attachment score only in the alpha frequency band and only over the frontal and parietal regions. In addition, behavioral results suggest that the present findings are not related to differences in the number of eye blinks between the two anxious attached groups (i.e., those in the A and T conditions) or possible correlation biases between anxious and avoidant attachment scales. Finally, we found no moderating effects of avoidance attachment style on the cortical brain activity between the two resting-state sessions.

It is vital to make a clear distinction between processes such as tonic alertness on the one hand and arousal and selective attention on the other (e.g., Oken et al., 2006). Arousal and selective attention are phasic reactions to specific stimuli, while tonic alertness is associated with nonselective readiness for perception and action, which plausibly occurs in the absence of any goal-directed task (Sturm and Willmes, 2001). Past research reports negative correlations between activity in regions associated with the regulation of selective attention processes and alpha power (Laufs et al., 2003; Capotosto et al., 2009). More recent studies confirm these results and suggest that alpha synchronization over posterior brain regions in resting state might imply enhanced tonic alertness (Sadaghiani et al., 2010). Based on the result of the previously mentioned studies and considering the task-free resting state procedure implemented in our work, we believe that the present findings suggest increased tonic alertness is required for more active introspective processes in the T compared to the A condition, which is reflected by enhanced alpha synchronization, high over posterior regions. We further found this conditional effect to be more strongly pronounced for the high anxious compared to low anxious participants. Those high vs. low in anxious attachment fail to have their need for approval met and become preoccupied with what other people might think about them when seated in silence beside another participant. Most importantly, however, this moderating effect of the anxious attachment style on the power of different EEG frequency bands during resting states in different social contexts was supported only for the alpha frequency band (frontal and parietal regions) which correlated highly with the participant's anxious attachment scores.

Interestingly, prior studies report that resting state with eyes open might involve some physiological changes in brain activity, such as increased functional connectivity in the DMN (Yan et al., 2009; Chen et al., 2013) and changes in synchronization patterns (Kuhnert et al., 2012). Non-verbal interactions, such as the simple mere presence of another person in close proximity, might be an essential and necessary condition for manifestation of attachment communication and attachment style activation. Mere presence with eyes open compared to eyes closed might create a more realistic and ecologically valid setting for the experimental condition where constant awareness of the current physical proximity of the other person is fundamental. In addition to the tonic alertness interpretation of our findings, in a recent study, Fransson (2005) proposed that in the resting state the brain is naturally predisposed to switch automatically between two opposite states: internally oriented vs. externally oriented. This spontaneous process during an eyes open resting state is conceived as a basic, evolutionary survival mechanism, which might facilitate repeated suspensions of introspective and self-referential processes in order to reallocate more resources toward areas engaged in evaluating the external environment and responding appropriately to potential threats (Mo et al., 2013). This line of reasoning is strongly associated with the inhibition theory which might serve as a complementary interpretation to the previously discussed tonic alertness explanation of our results. More precisely, during an internally oriented resting state the enhanced alpha power for anxious attached people might provide protection for the internal information processing, such as might occur when one wonders “what the other person is thinking about me,” by blocking external interferences coming from surrounding sensory input. It seems plausible to expect that the gating mechanism described above will be put in action only in the resting state with eyes open, which further supports the decisions made regarding the present experimental design. In line with our reasoning, Palva and Palva (2007) and Knyazev et al. (2011) suggest that enhanced alpha activity during resting state is associated with inhibition of external sensory perception and reduced attention, which might reflect internal mental processes.

In the context of the main tonic alertness interpretation of our findings, we find analogies with EEG-based research on anxiety that is different from and shouldn't be confused with anxious attachment style. For instance, Knyazev et al. (2006) suggests that enhanced posterior alpha activity in high anxious people reflects an increase in unspecific attention, which is evidence of higher general vigilance, especially in uncertain or social situations low on feedback. Klimesch (1999) arrives at a similar conclusion that enhanced alpha activity is associated with higher personal reactivity or readiness to adjust to external changes during resting state condition. Thus, this higher alpha power should not be perceived as an indicator of active inhibition, but more as a state of preparedness of a certain network.

Alpha oscillations might reflect several different brain processes such as active inhibition and tonic alertness which are both plausible explanations of our findings. Even though a combination of both processes (active inhibition and tonic alertness) seems like the most apparently valid explanation of the present results, we believe that our findings reflect increased tonic alertness which is required for more active introspective processes or readiness to adapt to unexpected external alterations mainly because we find stronger empirical and theoretical evidence in support of this interpretation.

With respect to the enhanced beta power in T compared to A resting state condition, past research suggests a positive correlation between beta band activity and the intrinsic alertness network (Sadaghiani et al., 2010). Some studies report enhanced beta band activity to be associated with an active state of alertness rather than a more passive sustained tonic alertness (Kamiński et al., 2012). However, we do not elaborate on beta and theta band activity since we did not find them significantly correlated with our participants' attachment score.

### Limitations and future research

The limitations of our study and opportunities for future research deserve mention.

First, we analyzed the average absolute power values in different frequency bands between two resting-state sessions, namely placing the same person in an A condition vs. T condition. Investigating the temporal evolution and possible interactions between the resting-state conditions by means of Granger causality would be an obvious next step to undertake. However, this requires changes in the experimental design, such as longer resting-state time.

Second, adding and analyzing different biomarkers might reveal useful strategies for discovering subtle differences between participants in the different resting-sate conditions. For instance, we could have examined whether heart rate for anxious attached people is likely to show greater variability and become lower in the dual conditions (Schmidt et al., 1999).

Third, our participants were from an international university that attracts students from different nations and cultures. Cultural differences or similarities could have affected the interpersonal dynamics. In our sample, southern European students interacted with northern European students, and Dutch-Dutch pairs interacted as well. Future research could examine the effects of cultural differences on changes in the social context during resting states.

Finally, our sample included women only, but attachment styles may differ across gender (Del Giudice, 2011). Our study could be extended to males or a mix of both genders to investigate gender differences and cross-gender effects.

### Conflict of interest statement

The authors declare that the research was conducted in the absence of any commercial or financial relationships that could be construed as a potential conflict of interest.

## Appendix 1

Anxious Attachment Style Scale (Brennan et al., 1998).

1. I worry that others won't care about me as much as I care about them.
2. My desire to be very close sometimes scares people away.
3. I need a lot of reassurance that my partner loves me.

## Appendix 2

Avoidant Attachment Style Scale (Brennan et al., 1998).

1. It helps to turn to my romantic partner in times of need (Reversed measure).
2. I turn to my partner for many things, including comfort and reassurance (Reversed measure).
